# Which choice of therapy when many are available? Current systemic therapies for advanced hepatocellular carcinoma

**DOI:** 10.1002/hsr2.147

**Published:** 2020-01-29

**Authors:** Nicola Personeni, Tiziana Pressiani, Lorenza Rimassa

**Affiliations:** ^1^ Department of Biomedical Sciences Humanitas University Pieve Emanuele Italy; ^2^ Humanitas Cancer Center Humanitas Clinical and Research Center—IRCCS Rozzano Italy

1

For more than a decade, a multikinase inhibitor, sorafenib, has represented the only systemic treatment in patients with unresectable hepatocellular carcinoma (HCC). Since 2017, additional targeted agents with peculiar antiangiogenic profiles have dramatically expanded the therapeutic armamentarium beyond sorafenib. However, many questions remain regarding the optimal use of such agents, and these will need to be urgently appraised, in particular, when it comes to timing, response evaluation, and predictive markers.

HCC is the most frequently diagnosed primary liver cancer and arises within a cirrhosis background in nearly 90% of patients.^1^ The strong relationship between the underlying liver disease and HCC justifies the need for staging systems that consider both competing risks, given that their respective impact on patients' prognosis is substantial. On these premises, the Barcelona Clinic Liver Cancer (BCLC) staging system^1^ is the most accepted algorithm, linking each disease stage to data‐driven interventions. With respect to systemic therapies, the BCLC algorithm currently recommends their use in HCC patients with advanced disease stage (C stage), while locoregional treatments should be considered in patients with an intermediate stage (B stage).

Within the heterogeneous BCLC B stage, several controversies still concern the adequate timing for the transition from locoregional to systemic therapies. Whereas lack of response after two rounds of transarterial chemoembolization (TACE) or progression of previously treated lesions indicates locoregional treatment failures,^2^ in a real‐world setting, these findings do not necessarily lead to an anticipated shift toward a systemic strategy.^3^ On top of that, it appears that a sizeable fraction of BCLC C patients still receives upfront TACE,^3^ which may contribute to acute and chronic liver function deterioration,^4^ despite a clear indication for other treatment options. Therefore, the possibility to identify those patients who might benefit from earlier transitions to systemic treatments speaks to the crucial role played by multidisciplinary teams that convene surgeons, interventional radiologists, hepatologists, and medical oncologists.

Both the vascular endothelial growth factor (VEGF) and its cognate receptors (VEGFRs) are among the most studied and well‐known mediators of angiogenesis and are relevant for HCC growth and progression.^5^ In fact, observations reporting an association between high VEGF levels, tumor angiogenesis, and progression^5^ constitute the basis to further explore developmental therapies centered on the VEGF‐VEGFR axis in HCC.

Sorafenib has been the breakthrough therapy refining the standard of care for patients with advanced or intermediate‐stage disease who progress on TACE.^6^ More recently, lenvatinib was shown to be noninferior to sorafenib as first‐line therapy,^7^ thereby expanding the therapeutic scenario with an additional, globally accepted treatment option. According to most recent figures,^7^ approximately one‐third of patients undergoing a frontline treatment also receives further anticancer medications. In this latter setting, regorafenib, cabozantinib, and ramucirumab have been approved by the United States (US) Food and Drug Administration (FDA) and the European Medicines Agency in patients who received sorafenib,^8^, ^9^, ^10^ while no data are yet available for patients who received prior lenvatinib. Though caution is mandatory when performing cross‐trial comparisons, in responding patients, a second‐line treatment that follows frontline lenvatinib or sorafenib^11^ could be as beneficial as the sequence sorafenib‐regorafenib provided by the RESORCE trial.^12^ Importantly, for HCC patients undergoing two lines of treatment, the median overall survival (OS) now exceeds 20 months, and a delayed time to clinical deterioration was reported when lenvatinib or ramucirumab was compared to sorafenib^7^ or placebo,^10^ respectively. Significant gains in terms of quality‐adjusted life‐years and time without symptoms and toxicity were also observed after treatment with cabozantinib.^13^, ^14^

In the US, two immune checkpoint inhibitors (ICI), namely, nivolumab and pembrolizumab, have been approved by the FDA in a second‐line context on the basis of the CheckMate 040^15^ and Keynote‐224^16^ trials, respectively. Despite such promising data, more mature phase III trials investigating nivolumab vs sorafenib^17^ and pembrolizumab vs placebo^18^ did not demonstrate statistically significant improvements in terms of OS with single‐agent ICI. On the other hand, cabozantinib may expand the therapeutic armamentarium, being itself also a third‐line option for patients pretreated with sorafenib.

Provided that patients must always meet essential eligibility criteria such as a preserved liver function (ie, Child‐Pugh score A) and good clinical conditions, these drugs cannot be used interchangeably. While head‐to‐head comparisons do not exist (except for lenvatinib and sorafenib, which were compared in the frame of a noninferiority trial),^7^ safety profiles and specific criteria that drew the framework of their development in the respective pivotal trials might better inform the clinicians' decisions. For instance, data gained on lenvatinib are limited to patients whose tumor volumes are less than/equal to 50% of the liver and to those with no portal vein invasion at the main portal branch or invasion of the bile duct. Likewise, in compliance with strict parameters already outlined in the RESORCE study protocol,^8^ patients candidate to regorafenib must be sorafenib tolerant, implying that for sorafenib‐intolerant patients, other treatments are more appropriate. Ramucirumab is superior to placebo only in patients with alfa‐fetoprotein (AFP) levels ≥400 ng/mL, but falls short of expectations when lower levels are considered.^19^ Mounting evidence indicates that specific adverse events such as hand‐foot syndrome and hypertension are related to drug exposure^20^ and could be considered as surrogate markers of survival as long as patients are on treatment with sorafenib,^21^ lenvatinib,^22^ regorafenib,^23^ and cabozantinib.^24^ Similarly, decreases in AFP levels (so‐called “AFP response”) may lead to early identification of patients who benefit more from sorafenib,^25^ cabozantinib,^26^ regorafenib,^27^ and ramucirumab.^28^ Also, the unprecedented objective response rate (by modified RECIST criteria) observed in the REFLECT study^7^ raises the hypothesis of a prognostic correlation with OS that was retrospectively confirmed, irrespective of treatment arm.^29^

With regard to pretreatment predictive biomarkers, however, AFP still stands as the only one being available to select patients in daily practice, thereby making ramucirumab the first biomarker‐driven therapy in HCC.^10^ In fact, the search for biomarkers predictive of treatment benefit in HCC remains rather elusive, in particular when it comes to antiangiogenic drugs. As a result, thus far, the “one‐size‐fits‐all” approach remains actively pursued in the field of HCC clinical research.

Prior investigations of the SHARP trial demonstrated that plasma concentrations of VEGF and Angiopoietin‐2 are independent prognostic factors for survival, but none predicted a benefit from sorafenib over placebo.^30^ Similar findings were reported also in the REFLECT^31^ and in the CELESTIAL trials.^32^ In contrast, plasma analyses from the RESORCE study indicated five proteins potentially involved in the inflammation process being predictive of regorafenib efficacy, but not prognostic.^33^ More pragmatically, retrospective analyses based on clinical parameters showed that benefit from sorafenib could be somehow higher in patients without extrahepatic spread and in patients with hepatitis C virus.^34^

These latter observations may indicate the possibility to link etiology with genomic alterations that eventually lead to increased sensitivity during treatment with specific agents. In this respect, apart from aflatoxin B1 exposure, the Cancer Genome Atlas Research Network genomics did not show major impact of etiology on HCC mutational signatures.^35^ Nevertheless, we believe that molecular profiling should remain instrumental for enrichment approaches in future trials, but this should come along with an increased awareness of the usefulness of tumor biopsy in HCC.^36^, ^37^

On the other hand, given the multiplicity of targets identified, single‐agent therapies, including multikinase inhibitors and ICI, are unlikely to be beneficial for a majority of patients. In fact, on the basis of a robust preclinical rationale,^38^ current approaches under investigation entail combinations of ICI and antiangiogenics, and these will pave the way for new therapies for the treatment of advanced HCC. Indeed, the recently presented IMbrave150 study is the first phase III trial that demonstrated the superiority of such a combination, namely, atezolizumab and bevacizumab, compared to sorafenib in the first‐line setting.^39^

The current treatment landscape for unresectable HCC is finally becoming more diverse and complex; several systemic agents have been approved and others have recently proved active and will be approved in the near future. Specific adverse events and AFP response are surrogate endpoints for survival and must be taken into account in clinical practice. Furthermore, considering the multiple drugs available, the timely transitioning from locoregional to systemic therapy now becomes even more crucial than before, in order to allow patients to receive all available treatment options. A multidisciplinary approach is, therefore, essential to offer optimal treatment to each patient, and further data on treatment sequences are needed to select first‐line, second‐line, and beyond therapies. Finally, the identification of biomarkers remains fundamental to define and select the different patient subgroups. The collection of tumor samples and liquid biopsies is, therefore, essential, to allow for the identification and validation of these biomarkers and to further improve our knowledge and therapeutic results.

## CONFLICT OF INTEREST

N Personeni reports receiving lecture fees from AbbVie and Gilead and travel fees from ArQule. T Pressiani declares no conflict of interests. L Rimassa reports receiving consulting fees from Amgen, ArQule, Basilea, Baxter, Bayer, Celgene, Eisai, Exelixis, Hengrui, Incyte, Ipsen, Italfarmaco, Lilly, MSD, Roche, Sanofi, Sirtex Medical; lecture fees from AbbVie, AstraZeneca, and Gilead; and travel fees from ArQule and Ipsen. These sources played no role in study design; collection, analysis, and interpretation of data; writing of the report; or the decision to submit the report for publication.
